# Metastatic porocarcinoma: A case demonstrating objective response to pembrolizumab

**DOI:** 10.1016/j.jdcr.2024.01.038

**Published:** 2024-02-29

**Authors:** Camélia Benhayoun, Anne Bénédicte Duval Modeste, Clémence Tamarit, Pascal Joly, Raphaël Janela-Lapert

**Affiliations:** a Dermatologie, C.H.U de Rouen, Rouen, France; b Anatomopathologie, C.H.U de Rouen, Rouen, France

**Keywords:** anti-PD1, immunotherapy, metastatic porocarcinoma, partial remission, pembrolizumab

## Introduction

Porocarcinoma (PC) is a rare adnexal tumor that develops from the acrosyringium, the intraepidermal portion of the excretory duct of the eccrine sweat gland. It is a highly aggressive disease with a mortality rate of 67% at an advanced stage.^1^ It is rare and represents 0.005% to 0.01% of skin tumors.^2^ In nearly 50% of cases, it is a tumor that develops on a preexisting benign poroma. Contradictorily, the most common locations are not those of healthy eccrine glands (palmo-plantar) but mainly at the head and neck and lower limbs. These are tumors with a localized prognosis, with frequent recurrence and nodal involvement. There are also cases of distant metastases.

Several treatments have been tried in the absence of current guideline, but only few have shown notable distinction. Systemic treatment is based on chemotherapy (taxanes, cisplatin) with low efficacy. We report a case demonstrating the efficacy of treatment with anti-programmed cell death-1 (PD-1).

## Observation

**Fig 1:**
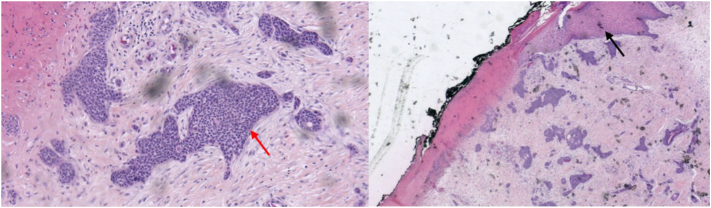
Histological sections with infiltrating and trabeculae; *Red arrow* shows basaloid cells and *black arrow* shows necrosis.

An 86-year-old patient presented a single left inguinal lymph node, which was found to be metastatic squamous cell carcinoma on histology. First-line treatment with 3 cycles of carboplatin was poorly tolerated and failed upon first reevaluation at 5 months. Due to the appearance of edema in the left leg and multiple inguinal lymph nodes, a reexamination of the initial slides and an excision of a recent lesion on the left lower limb was performed, revealing a tumor proliferation consisting of lobules and trabeculae infiltrating the dermis (Fig 1), composed of cells with a poral appearance, suggestive of metastatic cutaneous PC involving the lymph nodes. Considering the failure of carboplatin treatment, pembrolizumab, an anti-PD-1 therapy, was initiated in February 2022.

**Fig 2:**
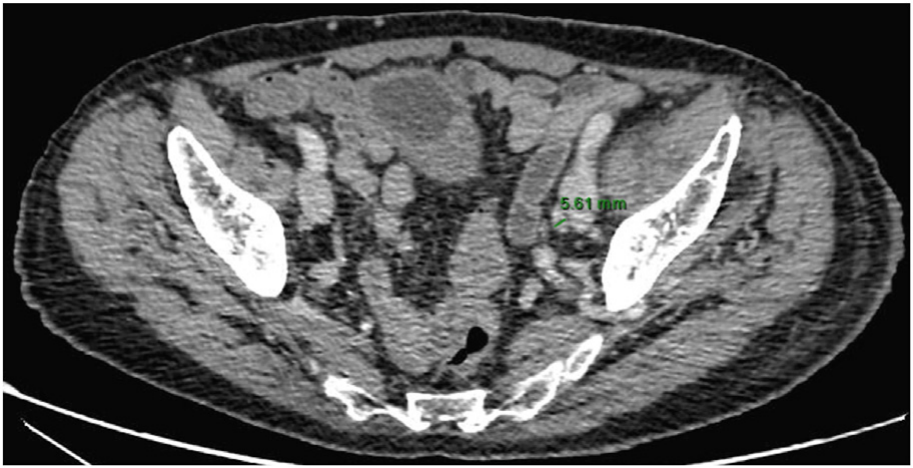
Axial computed tomography scan: regression of a left external iliac lymph node (13.69 mm to 5.61 mm from February to August).

**Fig 3:**
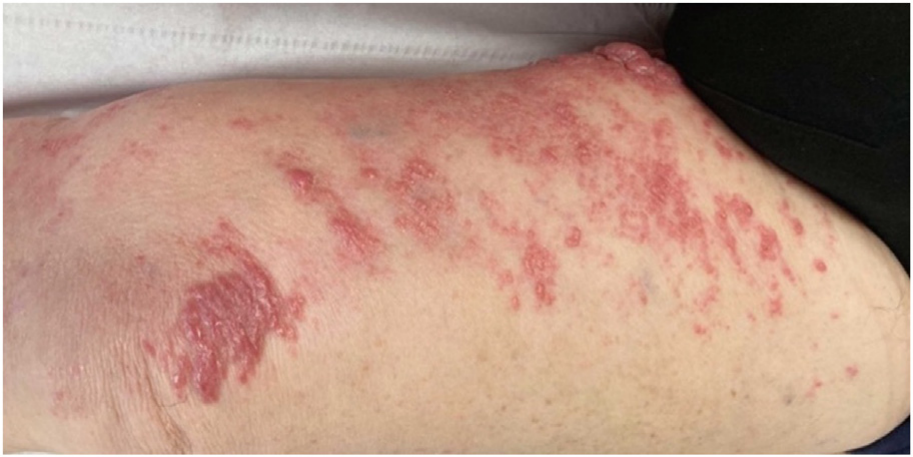
Nodular lesions of the left thigh before immunotherapy.

**Fig 4:**
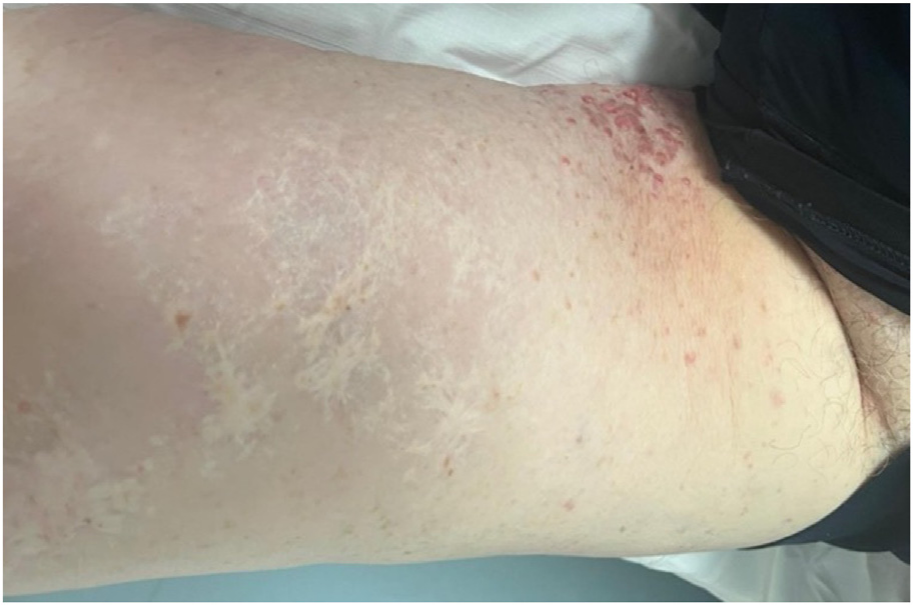
Objective clinical response after 12 months of pembrolizumab.

On the initial computed tomography scan, lymph node involvement was found in periclavicular, femoral, external iliac, and bilateral primitive iliac regions with left-sided predominance. After 5 months of treatment with pembrolizumab at a dose of 200 mg every 3 weeks, which was well tolerated, there was a significant regression of most lesions (partial remission) with disappearance of the previously described supra- and subdiaphragmatic lymph nodes (Fig 2) and partial regression of cutaneous nodular thickening in the external genitalia area with persistent lymphedema but with less discomfort (Figs 3 and 4). Retrospective immunohistochemistry evaluation of the skin biopsy in our patient showed an absence of PD1 marker (0%) and less than 1% of programmed cell death ligand-1 (PD-L1) marker.

This patient achieved 1 year of progression-free survival with treatment in January 2023. At 13 months, the patient finally presented with limited new right inguinal lymph node involvement. After 18 months, the patient is still in partial remission.

The patient did not experience any adverse effects throughout the entire duration of the treatment.

## Discussion

Anti-PD-1/PD-L1 antibodies have revolutionized cancer treatment by preventing cancer cells from suppressing the immune response. These treatments changed management of melanoma and Merkel cell carcinoma. Anti-PD-1 antibodies like cemiplimab and pembrolizumab are now approved for treating advanced nonmelanoma skin cancers, due to their high mutational burden, including cutaneous squamous cell carcinoma and basal cell carcinoma. These agents enhance anti-cancer T-cell activity, providing more durable responses and making PD-1 blockade the standard of care for certain advanced skin cancers.

We report a case of metastatic PC treated effectively with anti-PD1 and achieving an objective response. This result is promising in a pathology with a 30% risk of metastases, with over 50% being lymph node involvement.^3^

Until now, no treatment has demonstrated efficacy. Mohs surgery remains the gold standard. There are studies highlighting the efficacy of local treatments at an early stage, such as the application of imiquimod and maxacalcitol (a vitamin D derivative)^4^ or the use of intralesional interleukin 2^5^ or photodynamic therapy.^6^

At the metastatic stage, radiotherapy and chemotherapy such as taxanes, anthracycline, or platinum salts are widely used but with uncertain results. One study shows the effectiveness of cetuximab in PC based on the expression of epidermal growth factor receptor in the tumor but, unfortunately, the patient did not survive.^7^ In our case, immunohistochemistry revealed epidermal growth factor receptor expression on tumor cells, but we did not have enough evidence in favor of cetuximab to use it in the elderly patient.

To our knowledge, only 2 cases have been reported using pembrolizumab.^8^^,^^9^ The first case, reported in 2019, involved a 67-year-old female patient with PC on the left leg. She achieved complete remission after 15 months of treatment with pembrolizumab, following radiotherapy and 12 cycles of carboplatin with capecitabine. The biopsy showed positive immunoreactivity for PD-L1. The second case describes a 70-year-old male patient with a scalp skin lesion. Despite 3 Mohs surgical procedures and radiotherapy, he experienced recurrence and lymph node metastasis. Pembrolizumab was initiated leading to remission after 14 months of treatment.

In our case, as observed the literature, PC is frequently misdiagnosed and treated as squamous cell carcinoma. Unlike the other 2 patients, ours is the first presenting an objective response without prior radiotherapy. Additionally, our patient did not show any PD-L1 expression on the tumor. Just like in melanoma, the level of PD-L1 expression appears to be unrelated to the therapeutic response level.^10^ However, it is likely that other factors may play a role in the effectiveness of anti-PD-1 therapies. The lack of a direct correlation tumor PD-L1 expression may be explained by tumor heterogeneity (variable expression of PD-L1 within the tumor, resembling a mosaicism that can falsely be negative depending on the analysis location), the involvement of other immune checkpoints, the role of tumor mutational burden, as well as individual host factors. These theories underscore the complexity of immune interactions and suggest that the effectiveness of anti-PD-1 therapy depends on multiple factors beyond PD-L1 expression.

## Conclusion

We report a new case of efficacy treatment of a metastatic PC with anti-PD1. The efficacy of this treatment does not seem to be related to the PD-L1 expression but will need further explorations.

## Conflicts of interest

None disclosed.
